# The Roles of Four Novel P450 Genes in Pesticides Resistance in *Apis cerana cerana* Fabricius: Expression Levels and Detoxification Efficiency

**DOI:** 10.3389/fgene.2019.01000

**Published:** 2019-11-15

**Authors:** Weixing Zhang, Yufeng Yao, Hongfang Wang, Zhenguo Liu, Lanting Ma, Ying Wang, Baohua Xu

**Affiliations:** College of Animal Science and Technology, Shandong Agricultural University, Tai´an, China

**Keywords:** cytochrome P450 monooxygenases, Apis cerana cerana Fabricius, RNA interference, abiotic stresses, pesticides resistance

## Abstract

Cytochrome P450 monooxygenases (P450s) are widely distributed multifunctional enzymes that play crucial roles in insecticide detoxification or activation. In this study, to ascertain the molecular mechanisms of P450s in the detoxification of Chinese honeybees, *Apis cerana cerana* Fabricius (*A. c. cerana*), we isolated and characterized four new P450 genes (*Acc301A1*,* Acc303A1*,* Acc306A1, *and* Acc315A1*). The open reading frames of the four genes are 1263 to 1608 bp in length and encode four predicted polypeptides of 499 to 517 amino acids in length. Real-time quantitative PCR (RT-qPCR) results showed that expression of all four genes was observed in all developmental stages. In addition, Western blot assays further indicated the RT-qPCR results that showed that the four genes were induced by pesticide (thiamethoxam, deltamethrin, dichlorovos, and paraquat) treatments. Furthermore, we also used double-stranded RNA-mediated RNA interference to investigate the functions of *Acc301A1*,* Acc303A1*,**and* Acc306A1* in the antioxidant defense of honeybees. RNA interference targeting *Acc301A1*, *Acc303A1*, and *Acc306A1* significantly increased the mortality rate of *A. c. cerana* upon pesticide treatment. These results provide important evidence about the role of the four P450 genes involved in detoxification.

## Introduction

Organisms have a variety of antioxidant enzymes and detoxifying enzymes, including glutathione S-transferases (GSTs), carboxylesterases, and cytochrome P450 monooxygenases (P450s), that reduce the potential damage of environmental pollutants (Li et al., 2007). P450s are a supergene family that is found in all living organisms (bacteria, plants, fungi, and animals) and catalyzes a variety of oxidative reactions in the metabolism of endogenous and exogenous substrates (Feyereisen, 2005; Hannemann et al., 2006; Omura, 2010). Previous studies proved that P450 genes are involved in the bioconversion of chemicals from host plants and in the metabolism of a series of pesticides, such as pyrethroids, organophosphates, and carbamates (Li et al., 2007; David et al., 2013; Karunker et al., 2009). P450s are classified into different clades on the basis of their evolutionary relationship (Moktali et al., 2012). In insects, the P450 genes are classified into four major clades: the mitochondrial P450s (Mit P450s), CYP2, CYP3 (including CYP6 and CYP9), and CYP4. Nevertheless, we know little about the specific functions of the P450 genes in insects. The CYP3 clades, particularly those of the CYP6 and CYP9 families, have frequently been shown to play a role in the detoxification and the metabolic resistance to insecticides across a range of insect species (Liu et al., 2010; Poupardin et al., 2010; Cifuentes et al., 2012; Musasia et al., 2013).

Previous research has found that the overexpression of P450 genes is associated with insecticide resistance in many insects (Daborn et al., 2002; Nikou et al., 2003; Pridgeon et al., 2003). In* Tribolium castaneum*, the double-stranded RNA (dsRNA)-mediated knockdown of the expression of *CYP6BQ9* showed that *CYP6BQ9* confers deltamethrin resistance (Zhu and Snodgrass, 2003). Similarly, in *Drosophila melanogaster*, the overexpression of *Cyp12a4* provides resistance to lufenuron (Bogwitz et al., 2005). In resistant *Anopheles mosquitoes*, the two P450 enzymes, *CYP4G16* and *CYP4G17*, are frequently overexpressed and particularly highly enriched in the abdominal integument (Ingham et al., 2014). Early studies provided evidence that the *CYP4G11* gene plays an important role in protecting honeybees from insecticide (phoxim, paraquat, decamethrin) damage (Shi et al., 2013). The P450s in the honeybee genome play a crucial role in the detoxification of phytochemicals and pesticides in the diet (nectar, honey, and pollen) of both larval and adult stages (Mao et al., 2009). Also, Manjon et al. demonstrate that the CYP9Q family of both honeybees and bumble bees plays crucial roles in determinants of bee sensitivity to insecticide class (Manjon et al., 2018). In addition, like most other organisms, the honeybee P450s were involved in the detoxified of aflatoxin B1 (Niu et al., 2011).

*Apis cerana cerana* (*A*. *c*. *cerana*) has 52 P450 genes, with a similar number of genes found in *Apis mellifera* (Balfanz et al., 2012; Diao et al., 2018). It is a pity that there is limited knowledge of the functions of P450s, although the roles of P450s have been explored in other species. As a pollinator of flowering plants, *A. c. cerana* plays an essential role in maintaining the balance of regional ecologies and in agricultural economic development. *A. c. cerana* has a series of advantaged biological characteristics over *A. mellifera* such as high disease resistance and cold tolerance and the capability to fly long distances (Li et al., 2012a; Li et al., 2012b; Diao et al., 2018); forager workers of *A. c. cerana *are adept at collecting nectar from scattered floral resources, which are often neglected by forager workers of *A. mellifera* (Diao et al., 2018). However, in recent decades, the population size of *A. c. cerana* has obviously decreased in some regions, which is attributed to an epidemic of honeybee diseases and to the deterioration of its environment (Potts et al., 2010; Gegear et al., 2006; Kulhanek et al., 2017). Thus, it is crucial to identify the functions of P450s and to explore stress response mechanisms at the gene expression regulation level in *A. c. cerana*.

P450-mediated insecticide metabolism is an important mechanism involved in insecticide resistance. However, the molecular mechanisms of P450s in insecticide detoxification remain largely unknown, especially in honeybees. Currently, this topic has become a major focus and has led to widespread efforts to understand the molecular basis that underlies insecticide resistance. Previous researches identified and characterized several P450 genes and verified that those P450 genes responded to insecticide detoxification in *A. c. cerana* (Shi et al., 2013; Zhang et al., 2018). In this study, four novel P450 genes were identified and characterized. The gene structures were analyzed, and the phylogenetic tree of the four genes was established. We also examined the expression of four genes in different developmental stages. In addition, the analysis of real-time quantitative PCR (RT-qPCR) analysis suggested that the transcription levels of *Acc301A1*, *Acc303A1*, *Acc306A1*, and *Acc315A1* were upregulated with many insecticides (dichlorovos, thiamethoxam, deltamethrin, and paraquat). Our results provide preliminary insight into the changes in the gene transcription of *Acc301A1*, *Acc303A1*, *Acc306A1*, and *Acc315A1* and their responses and resistance to four insecticides (dichlorovos, thiamethoxam, deltamethrin, and paraquat). Western blot analysis further proved that Acc301A1, Acc303A1, Acc306A1, and Acc315A1 were upregulated by some insecticides at the protein level. The RNAi-induced gene suppression indicated that silencing of *Acc301A1*, *Acc303A1*, and *Acc306A1* repressed the transcriptional profiles of several stress response-related genes and increased the mortality rate of *A. c*.* cerana* under thiamethoxam treatment. Our results should be useful to facilitate further studies on the roles of P450 genes in pesticide resistance in insects.

## Materials and Methods

### Insects and Treatments

Animal housing facilities and handling protocols were approved by the Animal Welfare and Health Committee of Shandong Agricultural University. We procured honeybees from six healthy hives of an experimental apiary (Shandong Agricultural University, Tai’an, China). To analyze gene expression at different stages, samples were randomly collected from larvae (L1–L5, from the first to fifth instars), pupae (Pp: prepupae, Pw: white-eyed pupae, Pb: brown-eyed pupae, and Pd: dark-eyed pupae), and newly emerged adults (NE) (Michelette and Soares, 1993). Samples were frozen immediately in liquid nitrogen and stored at −80°C until use.

Throughout the experiment, foraging honeybee workers (they are estimated to be 20–35 days old) were randomly selected from the six colonies, and the selected honeybees were randomly placed into 30 wooden cages (dimensions of 10 × 7 × 8 cm), which were maintained in an incubator [33 ± 1°C, 60% ± 10% relative humidity (r.h.)]. Each treatment included six replicates, with 60 honeybees in each replicate. All the honeybee workers were starved for 6 h before provided with a diet. The control group was allowed free access to a diet of 50% (wt/wt) sucrose and water (Malone and Burgess, 2001), and the treatment groups were provided with a 50% sucrose solution with the different pesticides. Four different pesticides (dichlorovos, deltamethrin, thiamethoxam, and paraquat) were applied. The effective concentrations for each experimental group of the pesticides are shown in **Supplementary Table 1**. Samples were randomly obtained at the appropriate times (0, 3, 6, 12, and 24 h) and stored at −80°C after being immediately frozen in liquid nitrogen.

### Primers and PCR Amplification Conditions

The Primer 5 (Premier Biosoft International, USA) software was used to design the primers to amplify the open reading frame (ORF) and the promoter regions of *Acc301A1*, *Acc303A1*, *Acc306A1*,**and *Acc315A1*.**The sequences of the primers are listed in **Supplementary Table 2**. The GenBank accession number and PCR amplification conditions of all the genes used in the studies are listed in **Supplementary Table 2** and **Table 3**, respectively.

### RNA Extraction, cDNA Synthesis, and DNA Isolation

The total RNA from honeybees was isolated from samples stored at −80°C using the E.Z.N.A.^®^ Total RNA Kit II (OMEGA, USA) according to the manufacturer’s instructions. The PrimeScript™ RT reagent Kit with gDNA Eraser (TaKaRa, Japan) was used to generate first-strand cDNA according to the manufacturer’s instructions. Genomic DNA was isolated from workers with the QIAamp^®^ DNA Investigator Kit (QIAGEN, Germany) according to the manufacturer’s instructions.

### Cloning and Sequence Analysis of *Acc301A1*, *Acc303A1*, *Acc303A1*, and *Acc315A1*

The synthesized cDNA was used as the PCR template. All of the primers used in the study are shown in **Supplementary Table 2**, and the PCR amplification conditions are shown in **Supplementary Table 3**. PCR products were visualized on 1% agarose gels and purified using the Gel Extraction Kit (Solarbio, China). The purified products were cloned into pEASY-T3 vectors (TransGen Biotech, China) and transformed into competent *Escherichia coli* DH5 α (*E*.* coli* DH5 α) cells for sequencing by Sangon Biotech (Shanghai, China).

The isolation of the 5′-flanking region of *Acc301A1* and *Acc303A1 *was performed as previously described (Zhang et al., 2017). The primer sequences and PCR amplification conditions used in the study are shown in **Supplementary Table 2** and **Table 3**, respectively. The database TRANSFAC R.3.4 was used to search for transcription factor binding sites in the 5′-flanking region of *Acc301A1* and* Acc303A1* (the isolation of the 5′-flanking region of *Acc306A1* and *Acc315A1* failed).

DNAman version 5.2 (Lynnon Biosoft, Canada) was used to search for the ORFs of *Acc301A1*, *Acc303A1*, *Acc306A1*, and *Acc315A1 *and predict the theoretical isoelectric point (PI) and molecular weight of the proteins. Conserved domains in the four P450 genes were detected using bioinformatic tools available at the *National Center for Biotechnology Information* server (http://www.ncbi.nlm.nih.gov/Structure/cdd/cdd.shtml). Phylogenetic analysis was performed using Molecular Evolutionary Genetics Analysis 7 software (MEGA version 7) and the neighbor-joining method. The database of MitoProtII (http://ihg.gsf.de/ihg/mitoprot.html) was used to predict the potential mitochondrial-targeting peptides of the four P450 genes.

### Reverse Transcription Quantitative PCR

The expression levels of the four P450 genes were quantified using RT-qPCR using the ABI 7500 Real-time PCR System (Applied Biosystems, Foster, CA). The targeted genes and their specific primers for RT-qPCR are shown in **Supplementary Table 2**. We used β-actin (Gene accession no. XM_017065464) as the internal control for normalization and calculated the relative expression of mRNA in each of the treatments (**Supplementary Table 4**). RT-qPCR was performed on total RNA extracted from adult worker bees. The RT-qPCR was conducted in a 20-µL reaction, and the thermal cycling protocol was as follows: an initial denaturation at 95°C for 30 s, each cycle at 95°C for 5 s and 60°C for 34 s, for a total of 40 cycles. The fluorescence signal was measured at the end of each extension step at 60°C. After amplification, one dissociation step cycle of 95°C for 15 s, 60°C for 1 min, and 60°C for 15 s was performed to confirm that only the specific products were amplified. The relative expression levels of each gene were calculated* via *the 2^−ΔΔCt^ method (Livak and Schmittgen, 2001). To guarantee better reproducibility of the results, six biological replicates and three technical replicates were performed for all RT-qPCR analyses. Significant differences were analyzed using Statistical Analysis System version 9.1 software (SAS, USA).

### dsRNA Synthesis

Gene-specific primers (**Supplementary Table 2**) with a T7 polymerase promoter sequence (5′-TAATACGACTCACTATAGGGCGA-3′) at their 5′ end were synthesized for PCR amplification to synthesize dsRNA. The products were purified using a Gel Extraction Kit (Solarbio, China), and then the purified products were used to synthesize dsRNA with RiboMAX T7 large-scale RNA production systems (Promega, USA) according to the manufacturer’s instructions. DNase I was used to remove the DNA template of samples. Finally, the synthesized dsRNA was redissolved in RNase-free water and stored at −80°C until use. As a negative control, the dsRNA of the green fluorescent protein gene (GFP control; GenBank accession no. U87974) was also synthesized.

### Gene Silencing *via* RNA Interference

The newly emerged workers that were collected randomly from six colonies and divided randomly into six groups in wooden cages (n = 60/group) were used for RNAi experiments. In the treatment groups, workers were injected with 0.5 μL (10 μg/worker) of gene-specific dsRNA (ds301A1, ds303A1, ds306A1) using a microinjector (*Acc315A1* was not able to be silenced by dsRNA in our preliminary experiment). As the negative control, workers were injected with 0.5 µL of GFP dsRNA (GFP control) or 0.5 µL of sterile water (H_2_O control) or were injected with nothing (no injection control). The workers were kept in an incubator (60% r.h., 34°C, 24-h darkness regimen). At the appropriate time after injection, the honeybees were immediately frozen in liquid nitrogen and stored at −80°C until use. RT-qPCR was used to identify the three genes silenced in honeybees. RT-qPCR was also performed to detect the expression profiles of several detoxification enzymes, including *AccsHsp22.6*, *AccSOD2*, *AccTpx4*, *AccTpx1*, *AccGSTO1*, and *AccTrx1*, when *Acc301A1*, *Acc303A1*, and *Acc306A1* were silenced. Each experiment was repeated in six independent biological replicates.

### Enzymatic Activities of RNAi-Mediated Silencing Samples of *Acc301A1*, *Acc303A1*, and *Acc306A1*

The activity of superoxide dismutase (SOD), catalase (CAT), and peroxide (POD) in workers after injection with ds301A1, ds303A1, ds306A1 as the treatment groups and dsGFP as the control group (CK) separate at 12 or 24 h was measured with the SOD, CAT, or POD enzyme-linked immunosorbent assay (ELISA) kit (MIbio, China). Samples were homogenized in cell lysis buffer containing 10 mM phosphatase inhibitor (CWBIO, Beijing, China). The supernatants were recovered by centrifugation at 10,000*g *and 4°C for 10 min. Protein concentrations were quantified by a BCA Protein Assay Kit (Beyotime, Beijing, China). Next, ELISA analyses were carried out as described by Meng et al. (2018) to evaluate the activity of antioxidant enzymes. The optical densities of each well were measured with a Multiskan (BioTek Instruments, USA) at a wavelength of 450 nm. The activities of SOD, CAT, and POD were calculated according to the calibration curve, and the calibration curves are generated with the immunosorbent assay kit (ELISA) according to the manufacturer’s instructions. Each sample was run in three biological and three technological replicates. A one-way ANOVA was performed to analyze the differences between the control and treatment groups.

### Antibody Production

Custom-made polyclonal antibodies were used. An epitope was predicted using the GenScript Optimum Antigen design tool (the predicted peptide antigen of the four genes is shown in **Supplementary Table 4**), and the corresponding peptide antigen was then synthesized by Sangon Biotech (Shanghai, China). After the coupling reaction and mixing with complete adjuvant, the coupled antigen was used for injection. Next, the coupled antigen was mixed with incomplete adjuvant (Sigma, USA) and injected into five male Institute of Cancer Research mice (Taibang, China) that were six weeks old and pathogen-free, four times at 4-week intervals. The mice had free access to food (standard pellet diet) and tap water and were equally housed in a conventional steel cage with a 12-h light and dark cycles at room temperature. Subsequently, blood was collected by eyeball puncture and incubated at 37°C for 1 h, centrifugation at 3000*g* for 10 min. Finally, an anti-serum was prepared. The collected antibody was hybridized to a blot containing the total proteins to detect the specificity of the anti-serum.

### Western Blot Analysis

The samples were lysed in RIPA buffer (pH 7.5; Beyotime, Beijing, China), and then the lysate was centrifuged at 10,000*g* and at 4°C for 10 min according to the manufacturer’s instructions. Total protein concentrations were assessed using a BCA protein assay kit (Beyotime, Beijing, China). Next, equal amounts of protein per lane were separated by 10% sodium dodecyl sulfate polyacrylamide gel electrophoresis (SDS-PAGE) gels and subsequently transferred to a polyvinylidene difluoride membrane (0.22 µm; Millipore, USA) under 200 mA. The membrane was blocked with QuickBlock™ Western buffer (Beyotime, China) for 1.5 h prior to incubation with anti-Acc301A1, anti-Acc303A1, anti-Acc306A1, or anti-Acc315A1 primary antibodies at 4°C overnight. After washing three times in tris-buffered saline and tween (TBST) buffer (pH 8.0), the membrane was incubated with Horseradish peroxidase (HRP) -labeled goat anti-mouse IgG (H+L) secondary antibody (Beyotime, China) for 4 h at 4°C. Finally, the protein bands were detected using the enhanced BeyECL Plus kit (Beyotime, China) and visualized in Fusion Fx by a Vilber Lourmat. The detection of tubulin served as an internal control. The primary antibodies against Acc301A1, Acc303A1, Acc306A1, Acc306A1, Acc315A1, and tubulin were diluted at a ratio of 1:1000, and the secondary antibodies were diluted at a ratio of 1:1500. Each sample was run in three replicates. To quantity the protein levels, average spot intensities were measured for equivalent regions of interest using FusionCapt Advance FX7.

### Assessment of Mortality Rate

A total of 2400 honeybee workers were randomly assigned to 12 experimental groups with three cages per group. Treatment workers, which were injected with ds301A1, ds303A1, or ds303A, were compared to honeybees that were injected with dsGFP as the control (CK). Sample workers were fed a 50% sucrose diet containing dichlorovos, thiamethoxam, paraquat, or deltamethrin, respectively. **Supplementary Table 1** shows the dichlorovos, thiamethoxam, paraquat, and deltamethrin concentrations for workers. Then mortality was scored for 48 h, the dead honeybees were discarded, and the number of dead honeybees was carefully recorded in every group (Malone and Burgess, 2001).

### Statistical Analysis

One-way ANOVA was performed using Statistical Analysis System version 9.1 software (SAS, USA). When a significant treatment effect was detected, the Tukey *post hoc* test was used to determine differences between treatment groups. Equivalence of variance among groups was evaluated using Levene’s Test for homogeneity of variance. Data are presented as the means ±**SEM, and the value of *P *< 0.05 was considered statistically significant.

## Results

### Characterization of P450 Genes

In this study, we isolated *Acc301A1* (GenBank accession no. MK508995),* Acc303A1* (GenBank accession no. MK508998),* Acc306A1* (GenBank accession no. MK508996), and *Acc315A1* (GenBank accession no. MK508997) from *A. c. cerana*. The ORF of *Acc301A1*,* Acc303A1*,* Acc306A1*, and *Acc315A1 *ranged from 1263 to 1608 bp, encoding predicted polypeptides composed of between 499 and 517 amino acids, with predicted PIs from 8.48 to 9.07 and molecular masses ranging from 48.4 to 60.9 kDa (**Supplementary Table 5**).

As shown in **Supplementary Figures 1–4**, the characteristic active-site motifs, the putative hydrogen-bonding sequence Helix-K (ExxR), the heme-interacting region Helix-C (WxxxR), and the typical aromatic motif PXXFXP, which is part of the meander near the carboxyl end, were highly conserved among the four genes (Graham-Lorence and Peterson, 1996; Zhu and Snodgrass, 2003; Feyereisen, 2005; Nelson, 2006; Zhuang et al., 2011). In addition, the predicted *Acc301A1*,* Acc303A1*,* Acc306A1*, and *Acc315A1* genes share several characteristics with other members of the P450 supergene family, such as the characteristic signature motif of cytochrome P450 proteins FxxGxRxCxG (X represents any amino acid) (Tijet et al., 2001; Feyereisen, 2005; Nelson, 2006) (**Supplementary Figures 1–4**).

**Figure 1:**
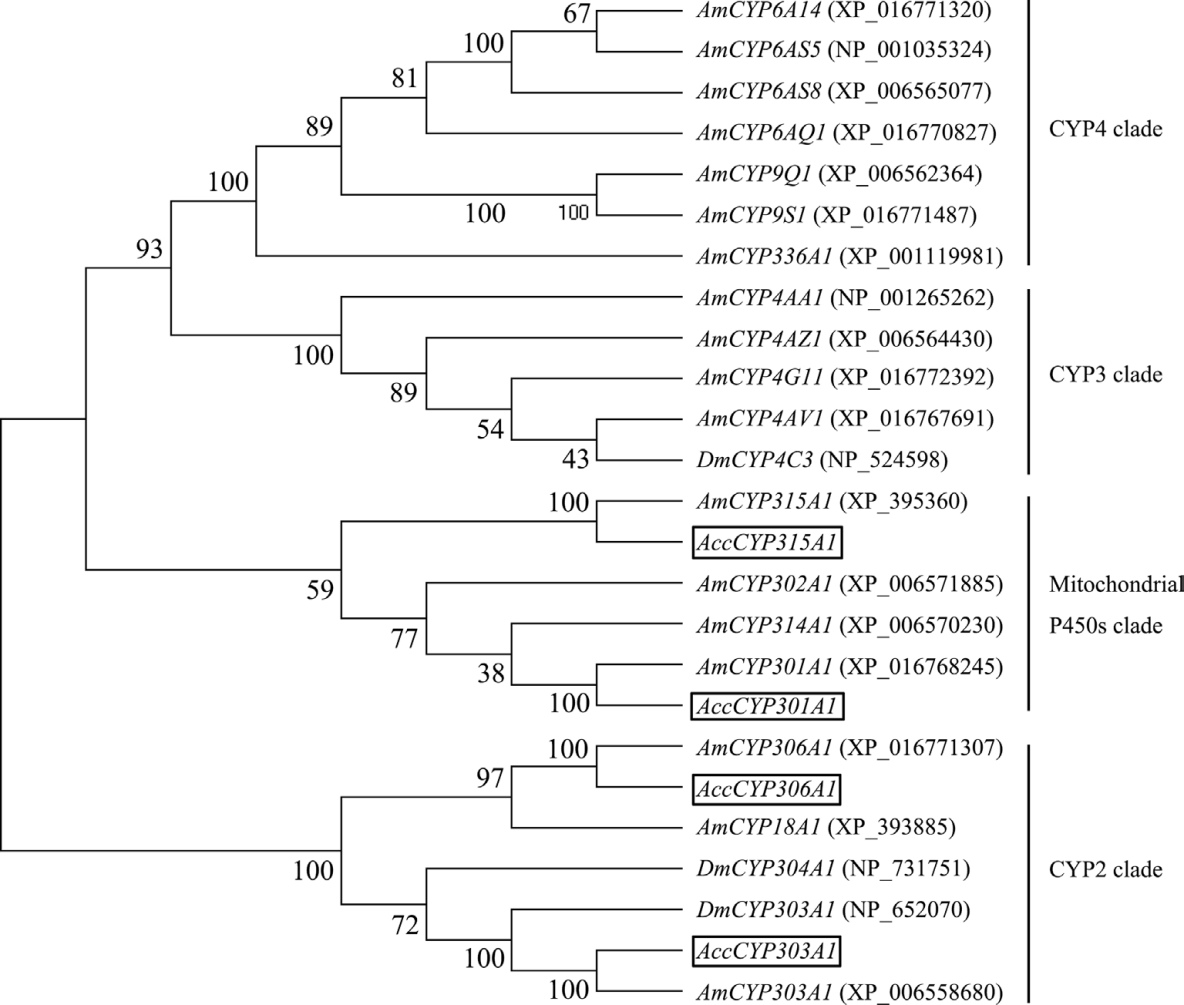
Phylogenetic tree of the four P450 genes. *Acc301A1*,* Acc303A1*,* Acc306A1*, and *Acc315A1 *were boxed.

The phylogenetic tree analysis showed that the deduced amino acid sequences of the four cloned genes were classified into two clades. Acc301A1 and Acc315A1 belong to the Mit P450s, and Acc303A1 and Acc306A1 were clustered into the CYP2 clade (**Figure 1**). Together, these results strongly demonstrate that the four genes were members of the P450 supergene family.

To better understand the potential roles of the P450 genes, the potential transcription regulator binding sites on 5′-flanking regions of the P450 genes are examined. Two fragments of *Acc301A1* and* Acc303A1* (1801 and 2038 bp, respectively) located upstream of the transcription start site were isolated (the isolation of fragments of* Acc306A1* and *Acc315A1* failed). The TFSEARCH website was used to predict the cis-acting elements of the 5′-flanking region of *Acc301A1* and* Acc303A1*. The TATA-box, which represents the putative core promoter element upstream of the transcription start site, was found. The cis-acting elements cell factor 2-II (CF2-II), Broad-Complex (BR-C), Caudal-related homeo-box (CdxA) transcription factors and NIT2 (**Supplementary Figure 5**) were identified and confirmed to be involved in development and various environmental stresses (Štanojević et al., 1989; Ericsson et al., 2006).

### Stage-Specific Expression

**Figure 2:**
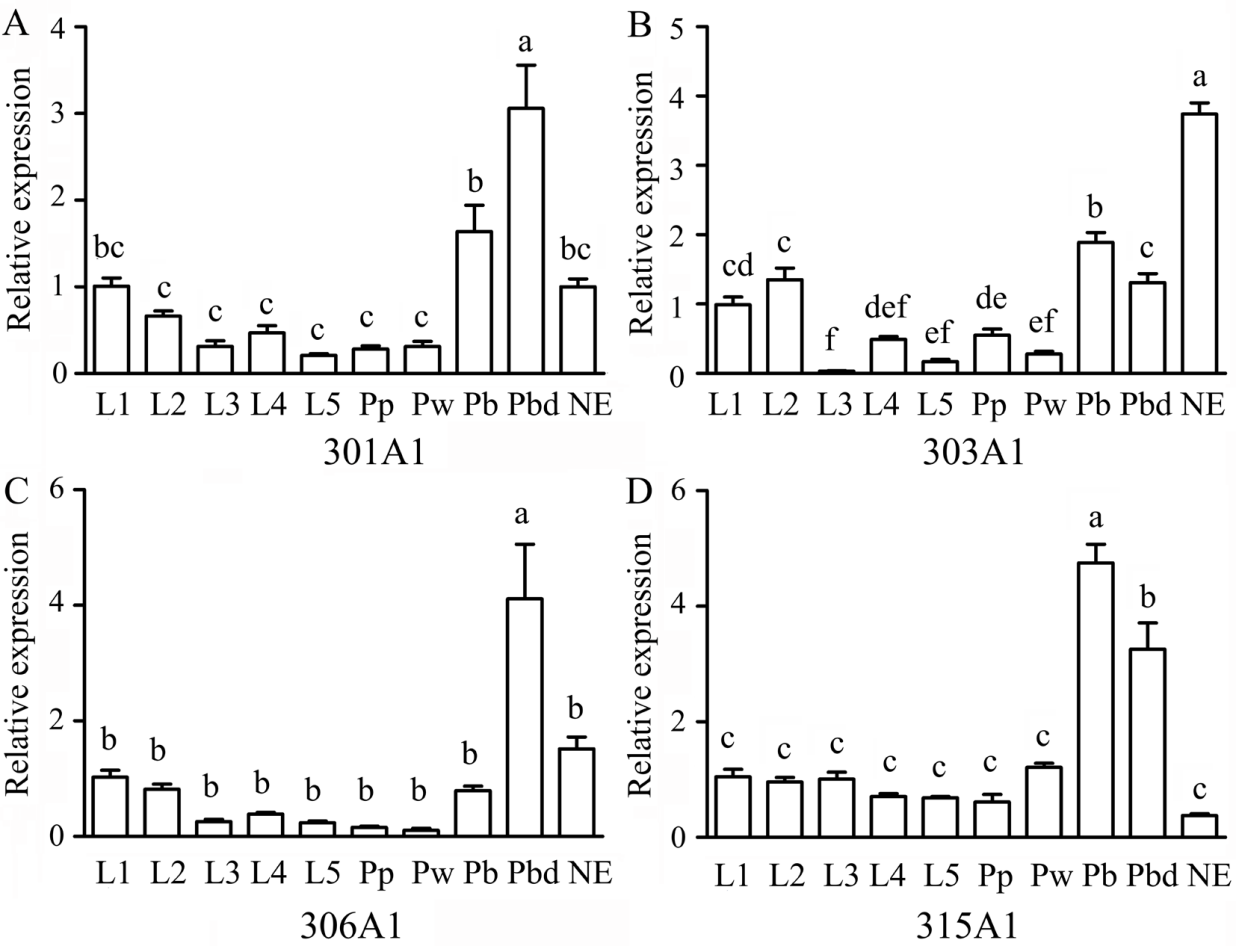
Expression levels of *Acc301A1* **(A)**,* Acc303A1* **(B)**,* Acc306A1* **(C)**, and *Acc315A1* **(D)** in different developmental stages by real-time quantitative PCR (RT-qPCR). Developmental stages: larvae (L1–L5), pupae (Pp, Pw, Pb, and Pbd), and adults (newly emerged workers) (n = 6). The β-actin gene was used as an internal control. Various letters above bars suggest significant differences between two groups (*P* < 0.05) based on one-way ANOVA followed by Tukey’s multiple comparison tests.

The RT-qPCR results showed that the relative expression profiles of *Acc301A1, Acc303A1, Acc306A1*, and *Acc315A1* were detected at all developmental stages. Overall, the expressions of all four genes in the larvae and the early stage of the pupa (Pp and Pw) were lower than those in the late pupa (Pb and Pbd) and NE (**Figure 2**), suggesting that they have specific functions in the late pupae and adults. Among the four genes, *Acc301A1* was found to be strongly expressed in Pbd in comparison with other stages (**Figure 2A**). As shown in **Figure 2B**, the expression of *Acc303A1 *decreased before increasing and peaking at NE. **Figure 2C** shows that *Acc306A1* is more strongly expressed in Pbd than in the other development stages. The transcription of* Acc315A1* showed no significant difference at the larvae and early pupae stages (Pp and Pw) and immediately increased and reached the peak at Pb before declining (**Figure 2D**).

### Effects of Insecticides on the Expression of P450s

**Figure 3:**
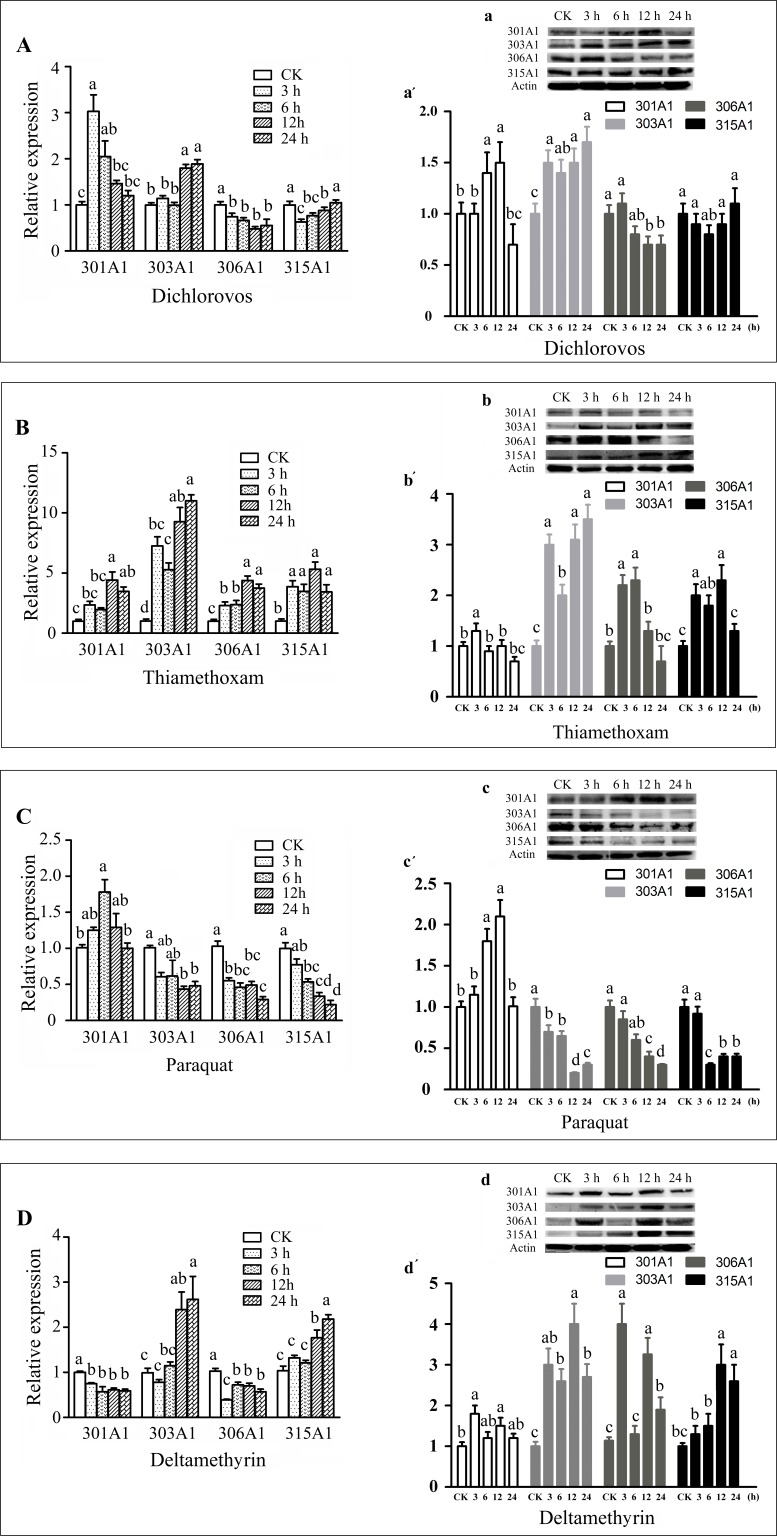
Expression profiles of the four P450 genes in adult workers under various abiotic stresses. These stresses are as follows: dichlorovos **(A)**, thiamethoxam **(B)**, paraquat **(C)**, and deltamethyrin **(D)**. Means ± SEM of three replicates of six individuals each. The β-actin gene was used as an internal control. Western blot analysis of the four P450 genes changes after they were treated with dichlorovos (a), thiamethoxam (b), paraquat (c), and deltamethrin (d); (a′, b′, and c′). Quantitation of protein levels. Equivalent quality of total protein from *A. c. cerana* was loaded for each sample.

In this study, we choose the four insecticides (dichlorovos, thiamethoxam, paraquat, and deltamethrin), which are the highly effective pesticides that are widely used in agriculture. Honeybee workers may contact insecticides when they forage for pollen and nectar outside. The effects of the four insecticides on the transcript and protein levels of *Acc301A1*,* Acc303A1*,* Acc306A1*, and *Acc315A1* in the honeybees were time dependent (**Figure 3**). As shown in **Figure 3A**, a, a′, when honeybees were treated with dichlorovos, the protein expression levels of *Acc301A1* and* Acc303A1* were induced and peaked at 12 and 24 h, respectively. Notably, the highest point protein expression level of *Acc301A1* compared with mRNA expression has a certain time delay, Interestingly, the protein level of *Acc303A1* reached its highest point rapidly at 3 h, while the highest transcript expression was at 12 h. However, the expression of *Acc306A1 *was slightly suppressed. The transcription of Acc315A1 was significant downregulated at 6 h, and then increased slowly until 24 h, in which no significant difference compared to the control group, while the protein expression level of Acc315A1 was not significantly different from the groups. When treated with thiamethoxam (**Figure 3B**, b, b′), the expression of all four genes increased significantly, while the protein level of 301A1 and 306A1 reached the highest point at 3 h, which is early than the peak of mRNA level at 12 h. In strong contrast, thiamethoxam treatment increased the protein level of 303A1 quickly already after 3 h, but the protein level decreased after 6 h and increased after 12 h of thiamethoxam treatment being significantly different from the control samples. The growth rate of the expression**of *Acc303A1* was higher than that of the other three genes at the peak. The mRNA expression trend is basically consistent with the protein level of 315A1. When honeybees were treated with paraquat (**Figure 3C**, c, c′), the protein expression of three of the four genes (*Acc303A1*,* Acc306A1*, and *Acc315A1*) declined, and the protein level of *Acc301A1* increased slowly, peaked at 6 h, and further increased after 12 h, albeit not significantly, whereas the expression quickly reduced after 24 h. The transcript expression levels of the four genes were basically consistent with the protein levels. As shown in **Figure 3D**, d, d′, the deltamethrin-induced downregulation of the mRNA expression of *Acc301A1* and *Acc306A1 *did not change significantly over time.**Notably, the protein levels of 301A1 and 306A1 was increased at 3 h, suggesting that the translation efficiency of mRNA has been improved when workers were exposed to deltamethrin. *Acc303A1* and *Acc315A1 *transcriptions were enhanced slowly by deltamethrin and reached the highest point at 24 h compared to the control group (2.62-fold and 2.18-fold, respectively), while at 3 h, the high protein level of Acc303A1 was observed suggesting high translation efficiency of Acc303A1. Deltamethyrin treatment increased the protein level of 315A1 quickly already after 12 and 24 h being significantly different from the control samples. Although there were several differences in the time points and the degree of expression, the protein expression trends of Acc301A1, Acc303A1, Acc306A1, and Acc315A1 were basically consistent with their transcript expressions (**Figure 3**). The above findings indicated that the four P450 enzymes might be involved in response to the four insecticides. In addition, to determine the specificity of antibodies, total protein was used to assess the specificity of anti-Acc301A1, anti-Acc306A1, anti-Acc306A1, and anti-Acc315A1 (**Supplementary Figure 6**). There is a single band near the protein’s molecular weight, suggesting that the antibodies were specific.

### Knockdown of Three Genes and the Expression Profiles of Other Antioxidant Genes

**Figure 4:**
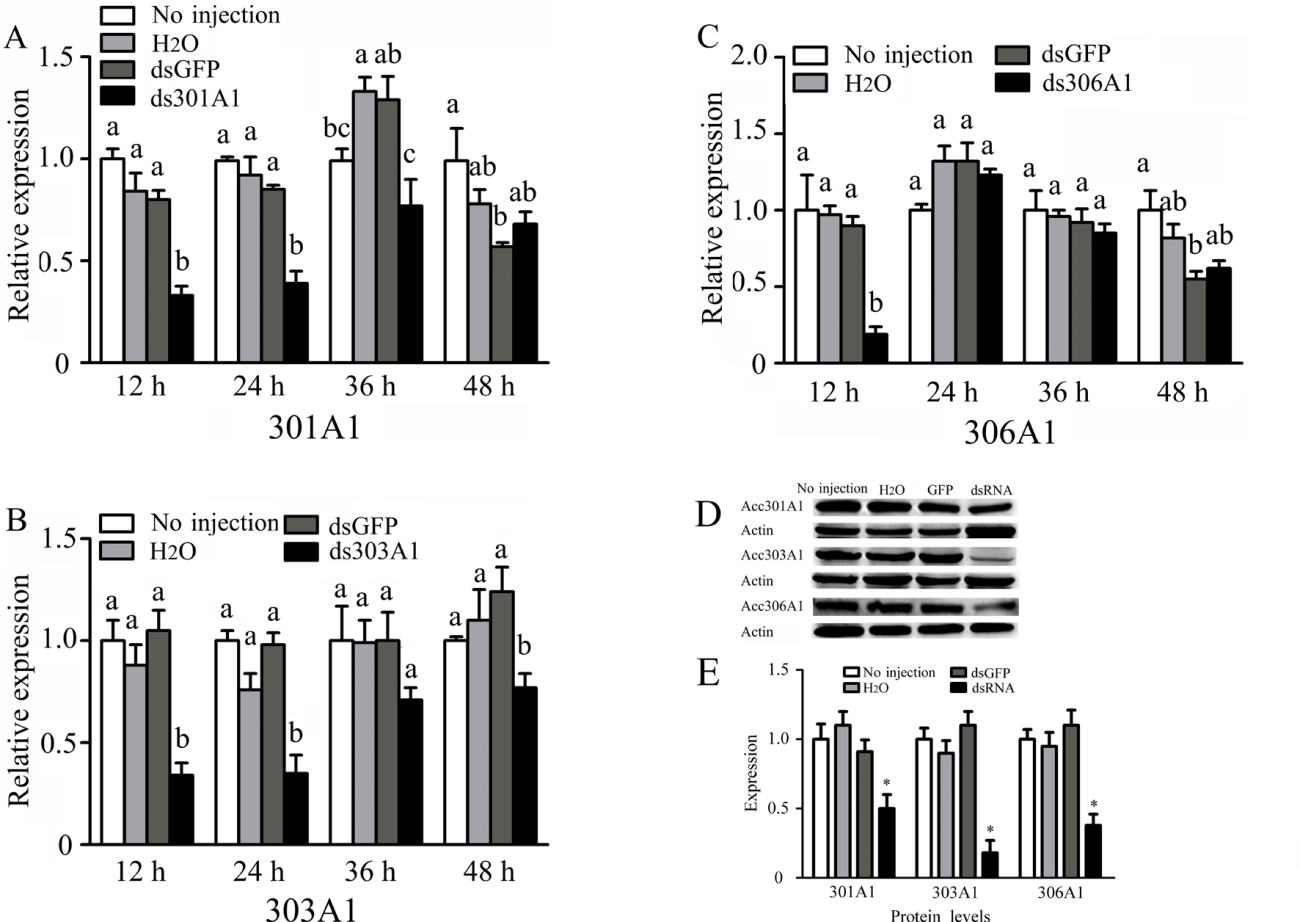
Effects of RNAi on mRNA levels of newly emergence honeybees, as induced by injecting 10 μg double-stranded RNAs. The mRNA levels of **(A)** *Acc301A1*, **(B)** *Acc303A1*, and **(C)** *Acc306A1* are shown. Western blot analysis of the three P450 proteins changes after one of the three P450 genes was silenced **(D)**. **(E)** Quantitation of Western blot analysis. Equivalent quality of total protein from *A. c. cerana* was loaded for each sample. RT-qPCR was performed on total RNA extracted from the samples. The β-actin gene was used as an internal control. Statistical significance of the gene expression among samples was calculated using one-way ANOVA followed by Tukey’s multiple comparison tests. Each value is given as the means ± SEM. Different letters or * above the bars indicate significant differences (*P* < 0.05).

To investigate the functions of *Acc301A1*,* Acc303A1*, and* Acc306A1* in the antioxidant defense of honeybee workers, RNAi experiments were performed (knockdown of Acc315A1 failed). As shown in **Figure 4**, RT-qPCR results show the transcription levels of *Acc301A1*,* Acc303A1*, and* Acc306A1* at different times (12, 24, 36, and 48 h) after injection of dsRNA against the three P450 genes. All the transcript levels of the three P450 genes showed maximum silencing at 12 h relative to the expression of the control groups. In addition, we compared the three protein levels of controls and the silenced groups using quantitative Western blot analysis (**Figure 4D, E**). For each group, we individually tested three workers and found that all of the three proteins are various degrees of repression in the silenced groups, respectively.

**Figure 5:**
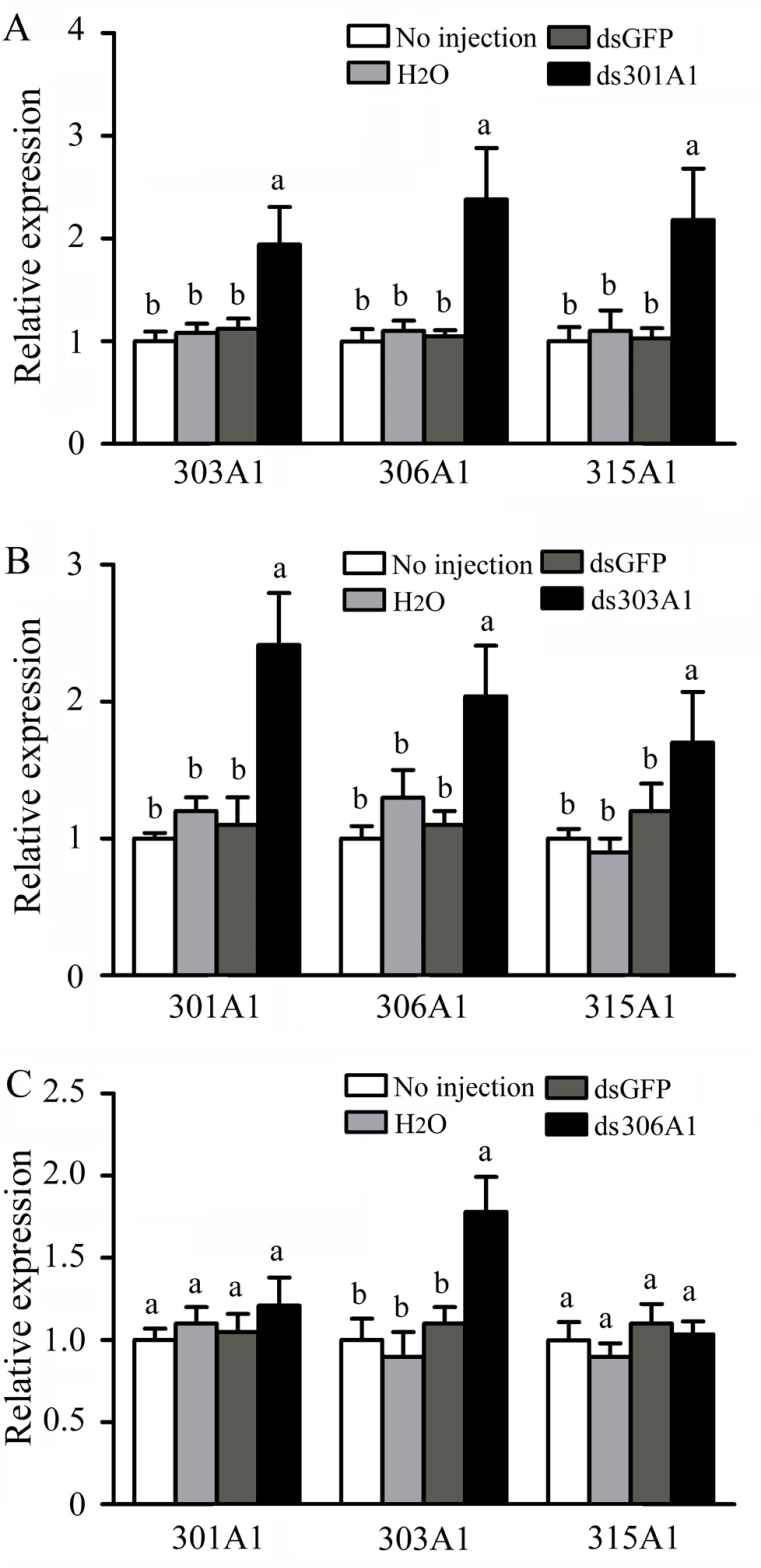
Transcription levels of other three P450 genes performed using RT-qPCR when **(A)** *Acc301A1*, **(B)** *Acc303A1*, and **(C)** *Acc306A1* were knocked down. The**β-actin gene was used as an internal control. Each value is given as the means ± SEM. Different letters above the bars indicate significant differences (*P* < 0.05), according to SAS software 9.1.

To evaluate the response of the other three P450 genes after one was silenced at 12 h, RT-qPCR was used. When *Acc301A1 *was silenced at 12 h, the transcription of *Acc303A1*, *Acc306A1*, and *Acc315A1* was induced (**Figure 5A**). As shown in **Figure 5B**, when *CYP303A1* was silenced, the transcription levels of *Acc301A1*,* Acc306A1*, and *Acc315A1* were enhanced. When *Acc306A1* was knocked down for 12 h, the expression level of *Acc303A1* was upregulated, while the expression levels of *Acc301A1* and *Acc315A1 *showed no significant differences (**Figure 5C**).

**Figure 6:**
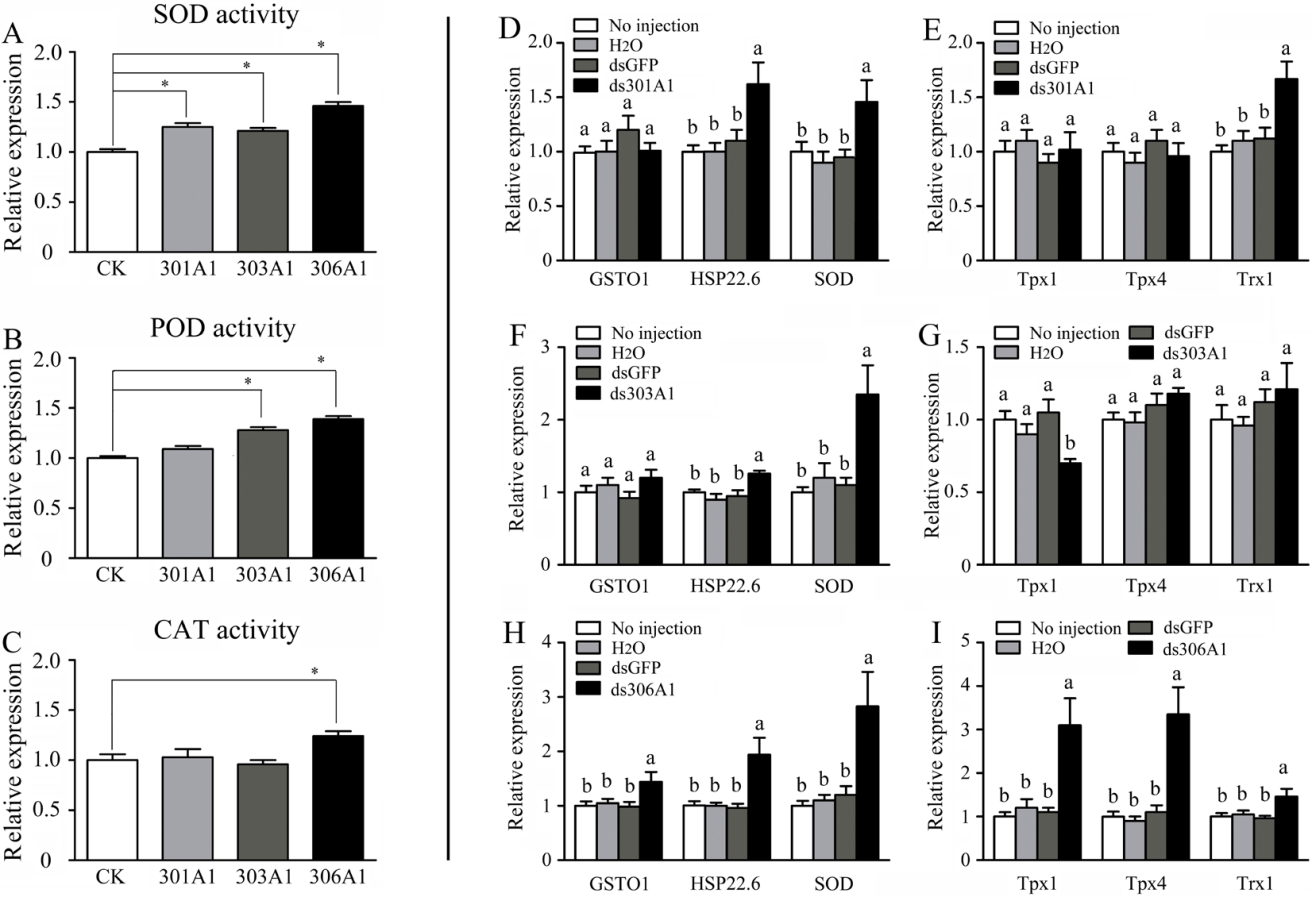
Effects of *Acc301A1*, *Acc303A1*, and *Acc306A1* knockdown separately on antioxidant enzyme activities in *A. c. cerana*. **(A)** SOD activity. **(B)** POD activity. **(C)** CAT activity. Relative mRNA levels of several stress response genes were investigated by RT-qPCR when silencing *Acc301A1* **(D**, **E)**, *Acc303A1* **(F**, **G)**, and *Acc306A1* **(H**, **I)**. Means ± SEM are given (n = 6). Significant differences between different groups were presented by various letters or * above the bars according to Tukey’s multiple comparison tests (*P* < 0.05).

When one of the P450 genes were silenced, in which honeybees will be exposed to a high level of oxidative stress, the honeybees might respond or adapt to such conditions by upregulating other antioxidant genes. To test whether this was the case, we determined the activities of several antioxidant enzymes (SOD, POD, and CAT), which had been demonstrated to play important roles in scavenging hydrogen peroxide (Corona and Robinson, 2006). Our results describing SOD activity are displayed in **Figure 6A**. When *Acc301A1*,* Acc303A1*, and *Acc306A1* were knocked down, the activity of SOD was significantly induced compared with that of the control group. Silencing* Acc303A1* and *Acc306A1* individually resulted in significantly enhanced POD activity in the silenced group compared with those of the control group. The activity of POD was the same in the control group and in the *Acc301A1 *knockdown group (**Figure 6B**). The activity of CAT was significantly higher than that of the control samples when *Acc306A1* was silenced. CAT activity did not show a significant increase compared with that of the control group when Acc301A1 and Acc303A1 were knocked down separately (**Figure 6C**).

The effects of RNAi of *Acc301A1*, *Acc303A1*, and *Acc306A1* on the transcription levels of several stress response genes that have been reported to be involved in oxidative stress responses (Meng et al., 2014; Yao et al., 2014; Zhang et al., 2014) were investigated using RT-qPCR. The β-actin gene was used as a reference gene. The transcription levels of *AccSOD*, *AccHSP22.6*, and *AccTrx1* were significantly upregulated when *Acc301A1* was knocked down (**Figure 6D**). The transcription of *AccTpx1* and *AccTpx4* was not significantly impacted when *Acc301A1* was silenced compared with the control groups (**Figure 6E**). When *Acc303A1* was silenced, the expression level of *AccSOD* was upregulated approximately 2.357-fold (**Figure 6F**), the expression levels of *AccGSTO1*, *AccHSP22.6*, *AccTpx4*, and *AccTrx1* were not significantly impacted compared with those of the control groups, and the transcription levels of *AccTpx1* were downregulated (**Figure 6G**). The knockdown of Acc306A1 increased the expression profiles of *AccGSTO1*, *AccHSP22.6*, *AccSOD*, *AccTpx1*, *AccTpx4*, and *AccTrx1* (**Figure 6H, I**) compared to the expression levels of controls. These findings indicate that the mRNA profiles of several stress response genes are influenced by the knockdown of Acc301A1, Acc303A1, and Acc306A1. Acc301A1, Acc303A1, and Acc306A1 may play important roles in the stress response.

### Mortality Assays

**Figure 7:**
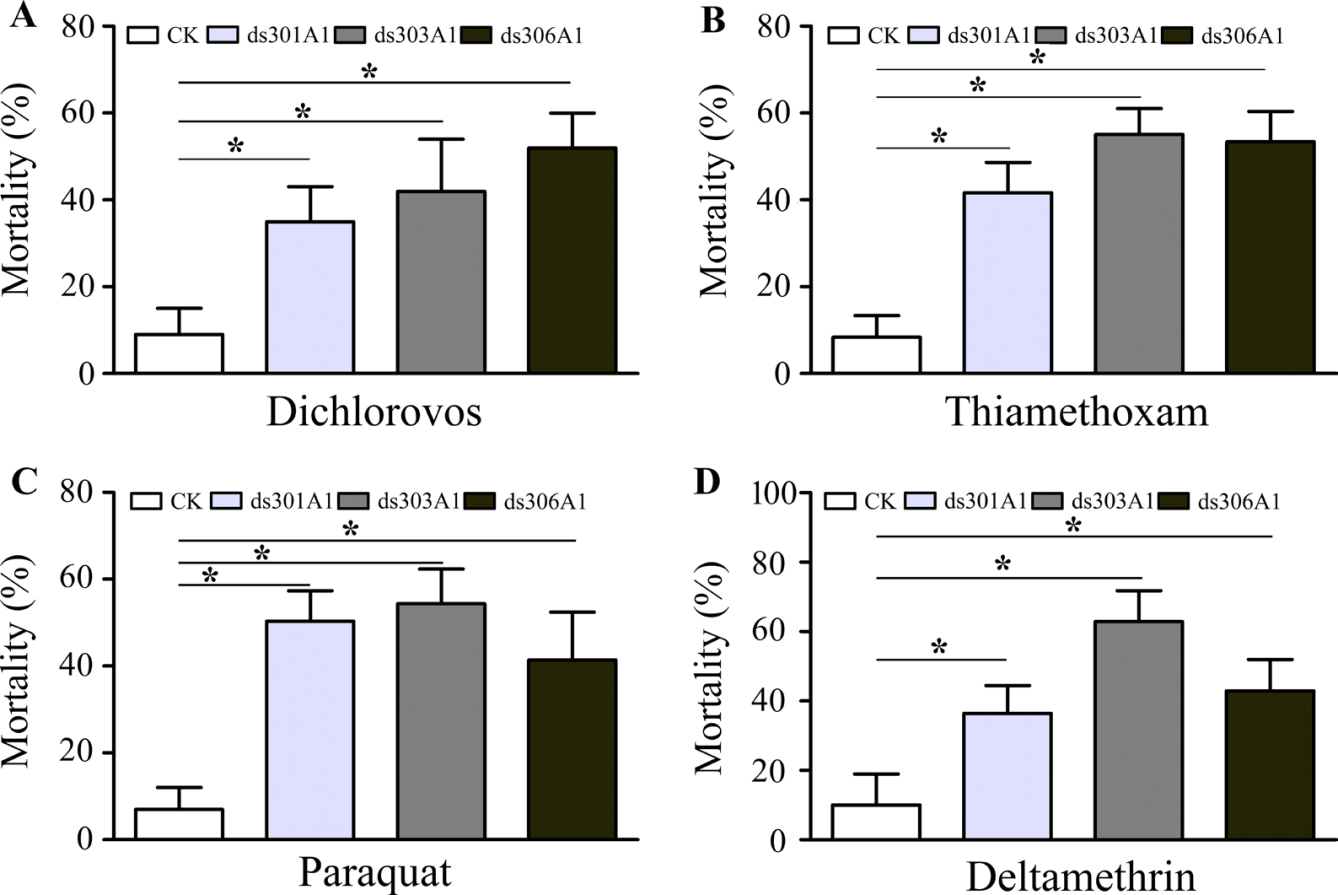
Mortality rate of honeybees after a 48-h exposure to dichlorovos **(A)**, thiamethoxam **(B)**, paraquat **(C)**, and deltamethrin **(D)** when knockdown of *Acc301A1*, *Acc303A1*, and *Acc306A1* was recorded. Means ± SEM of three replicates of 50 individuals each. Significant differences between different groups were presented by various letters or * above the bars according to Tukey’s multiple comparison tests (*P* < 0.05).

To obtain a view of the importance of P450 genes in response to pesticides, the P450 genes were silenced to examined the P450 genes for the response of four insecticides (dichlorovos, thiamethoxam, paraquat, and deltamethrin) by mortality assays. There was a significantly increased final percent mortality in honeybee workers injected with dsRNA treatments compared to the control group (**Figure 7**). When workers were treated with dichlorovos, there was a significant effect on mortality and increased mortality when honeybee workers were silenced: Acc301A1 (35%), Acc303A1 (42%), or Acc306A1 (52%) (**Figure 7A**). Mortality assays demonstrated that when workers were treated with thiamethoxam, the Acc301A1 (41.7%), Acc303A1 (55.1%), or Acc306A1 (53.3%) silenced groups enhanced the mortality in the workers (**Figure 7B**). As shown in **Figure 7C**, when honeybee workers were exposed to paraquat, mortalities were higher in the paraquat treatment groups than in the control groups (8% vs. 50.8%, 8% vs. 55.3%, 8% vs. 42.6%) in this study. As for deltamethrin, significant differences were observed in the silenced groups compared to the control groups (**Figure 7D**). Thus, the above results suggest that the P450 genes may play a critical role in resisting pesticide stresses.

## Discussion

In the present study, we isolated and characterized four novel P450 genes (*Acc301A1*, *Acc303A1*, *Acc306A1*, and *Acc315A1*) from *A. c. cerana*. The four genes belong to the Mit P450 and CYP2 clades. The RT-qPCR results showed the development stage-specific expression profiles of the four P450 genes. In addition, we demonstrated that the transcriptional levels of four P450 genes were regulated under different environmental stresses, and Western blot assays further supported our results. Furthermore, the knockdown of *Acc301A1*, *Acc303A1*, and *Acc306A1* induced the expression of several stress response-related genes and increased the mortality rate of honeybees under insecticide treatments. Our results thus reveal that *Acc301A1*, *Acc303A1*, *Acc306A1*, and *Acc315A1* may play important roles in the defense against adversity.

Phylogenetic analysis, on the basis of the amino acid sequences, showed that *Acc301A1*, *Acc303A1*, *Acc306A1*, and *Acc315A1* clustered with the separate Mit P450 and CYP2 clades. The phylogenetic analysis (**Figure 1**) and the observation of conserved characteristic functional domains (**Supplementary Figure 1–4**) suggested that the four P450 genes are indeed P450 supergene family members and had high similarities to four *A. mellifera *genes, *Am301A1*, *Am303A1*, *Am306A1*, and *Am315A1.* Thus, we named the four novel genes* Acc301A1*, *Acc303A1*, *Acc306A1*, and *Acc315A1*.

Transcription factors can serve as specific switches, which can turn sets of genes on or off at appropriate locations and at different times during development (Mitchell and Tjian, 1989). In this study, the 5′-flanking region of *Acc301A1* and *Acc303A1* contains many transcription factor binding sites involved in development and growth (**Supplementary Figure 5**). Previous evidence has demonstrated that Cdx plays critical roles in numerous developmental processes (Boudreau et al., 2002). A large number of studies supported that the transcription factor BR-C is involved in controlling insect growth and development by mediating ecdysone and juvenile hormone (Zhou and Riddiford, 2002; Uhlirova et al., 2003; Dubrovsky, 2005). To confirm whether the P450 genes are involved in development and growth, the transcription patterns of the four genes were analyzed in different developmental stages. The RT-qPCR analyses indicated that *Acc301A1*, *Acc306A1*, and *Acc315A1 *were expressed higher in the later pupae (Pbd, Pbd, and Pb, respectively), and the higher expression of *Acc303A1* was observed in NE (**Figure 2**). It was speculated that during periods of lifecycle transition, honeybees will increase metabolism to adapt for development, and the upregulated transcription levels of the four genes may help reduce toxic chemicals and oxidative damage (Cadenas and Davies, 2000; Alias and Clark, 2007; Guo et al., 2015). Interestingly, the transcription of *Acc301A1*,* Acc306A1*, and *Acc315A1* was detected obviously decreased in NE, implying that those genes might play other roles in insect physiology.

The extensive and repetitive use of pesticides in agriculture had led to a decrease in the population of honeybees, which is the most important pollinator of flowering plants (Liu et al., 2005; Elbert et al., 2010; Puinean et al., 2010; Gore et al., 2013; Chen et al., 2016). The cytochrome P450 enzymes involved in the detoxification of several xenobiotics have been investigated in several insect species (Guo et al., 2015). Studies on the P450s mainly focused on the vital roles in the insecticide resistance and metabolism of toxic chemicals and in the processes of biosynthesis or inactivation of endogenous compounds (Gonzalez, 1990; Durst and Benveniste, 1993; Zhou et al., 2010; Kola et al., 2015; Yu et al., 2015). In addition, it has been proven that metabolic detoxication mediated by P450s contributes significantly to honeybee tolerance of pyrethroid insecticides (Johnson et al., 2006). Therefore, it is particularly necessary to study the insecticide resistance in *A*. *c*. *cerana*. As above data, some transcription factor binding sites involved in environmental stimuli have been found in the 5′-flanking region of Acc301A1 and Acc303A1. Accumulated studies have shown that the transcription factors NIT2 (Tao and Marzluf, 1999) and HSF (Fernandes et al., 1994; Ding et al., 2005) may play important roles in response to various environmental stresses. To obtain a view of the transcriptional changes of P450 genes in response to pesticides, the four P450 genes were examined for the response of four insecticides (thiamethoxam, deltamethrin, dichlorovos, and paraquat) by RT-qPCR.

In the study, Acc301A1 was induced by dichlorovos, thiamethoxam, and paraquat treatments, and Acc303A1 was upregulated by dichlorovos, thiamethoxam, and deltamethrin treatments. Acc306A1 and Acc315A1 were stimulated by thiamethoxam and deltamethrin treatments. In this study, the transcriptional patterns and protein translation of Acc301A1, Acc303A1, Acc306A1, and Acc315A1 were different to various degrees and at different times (**Figure 3**) with different pesticides, indicating that their functions in response to different pesticides might be somewhat distinct. It has been proven that various xenobiotic-inducible P450s respond to different signaling pathways, suggesting the dynamic nature and diverse roles of the P450 genes in physiological pathways, drug metabolism/detoxification, and pathological processes (Su et al., 1990; Usui et al., 1990; McSorley and Daly, 2000; Nebert and Russell, 2002; Sangar et al., 2010). In addition, previous research showed that variation in honeybee sensitivity to neonicotinoids resides in divergent metabolism by P450s (Manjon et al., 2018).

These results provide evidences that the P450 genes may be responsible for insecticide resistance or the metabolism of the xenobiotics and pesticides. Of the four P450 genes identified and characterized in this study, the relative transcription levels of Acc301A1, Acc303A1, Acc306A1, or Acc315A1 were increased or suppressed under different pesticide treatments compared to that of the control groups. Therefore, the possible roles of the genes were further investigated using loss-of-function experiments. In case where one of the three P450 genes was silenced, the expression of the other genes was increased, except that no significant difference was seen in the transcription levels of Acc301A1 and Acc315A1 when Acc306A1 was silenced. This finding clearly demonstrates that the four P450 genes might have some kind of intrinsic link in insecticide resistance.

To further understand the mechanisms of metabolic resistance to different insecticides in *A. c. cerana*, the activities of three antioxidant enzymes (CAT, SOD, and POD), which play important roles in scavenging reactive oxygen species (ROS) that can cause oxidative damage to DNA, proteins, and lipids in an organism (Corona and Robinson, 2006), were determined after one of the three P450 genes was silenced. SOD catalyzes the dismutation of superoxide radicals to H_2_O_2_ and oxygen, and CAT reduces H_2_O_2_ to water and oxygen, protecting cells from oxidative injury (Ahmad et al., 1991; Kostron et al., 1999). Furthermore, the enzymatic activity of SOD, POD, and CAT, which are essential enzymes in insect, responses to ROS. Interestingly, the different P450 genes silenced, respectively, manifested different effects on the enzymatic activity of SOD, POD, and CAT. When one of the P450 genes was knocked down, the increased activities of antioxidant enzymes are compensatory physiological mechanisms in response to oxidative stress. However, there was no significant difference in POD activity between the ds301A1 group and the control group. In addition, when *Acc301A1* and *Acc303A1* were silenced, the enzymatic activity of CAT was not significantly increased compared with that of the control group. All the above-mentioned results indicate that the *Acc301A1*, *Acc303A1*, and *Acc306A1* were involved in abiotic stress responses and the transcriptional patterns of the P450 genes were different to various degrees.

GSTs and SODs are major families of detoxification enzymes that promote resistance to insecticides in insects. Previous research reported that GST could be induced by exposure to severe pesticides in many insects (Singh et al., 2001; Yu, 2004). *AccTpx1*, *AccGSTO1*, *AccTrx1*, and *AccsHSP22.6* were confirmed to be involved not only in response to different abiotic stresses but also in development (Yu et al., 2011; Meng et al., 2014; Yao et al., 2014; Zhang et al., 2014). Our results show that the expression patterns of antioxidant enzyme genes were different to some extent. The manner in which the silencing of one of the P450 genes differentially affected the expression patterns of different detoxification enzymes was complex. These results indicated that silencing one of the three P450 genes may exert different effects on the biological and biochemical responses of *A. c. cerana*.

To gain insight into the connection of the Acc301A1, Acc303A1, and Acc306A1 genes with pesticide treatments in* A. c. cerana*, RNAi-induced gene suppression was used. RNAi-induced gene suppression has been reported to be an effective strategy for silencing target genes involved in the metabolic activities of insects (Kumar et al., 2009; Huvenne and Smagghe, 2010; Naito and Ui-Tei, 2012; Gong et al., 2013). This study is identifying the specific P450 genes contributing to pesticide metabolism in honeybees, which is facing pesticide exposures when collecting nectar and pollen in agricultural fields. The results of the study clearly show that independent injection with dsRNA targeting *Acc301A1*, *Acc303A1, *and *Acc306A1 *increased the mortality rate of *A. c. cerana* under pesticide stress (**Figure 7**), suggesting that the three P450s may play essential roles in the survival of insects in response to pesticide stress. Different mortalities among the four insecticides may be caused by a different tolerance of pesticide-induced stress in honeybees. It is also likely that the different mechanisms in the insecticide resistance of P450s may play a role in mediating the reduced toxicity to honeybees. Perhaps this results from differences in their efficiency of metabolism by different P450 genes. Similar results were also observed when larval survival rate and growth were reduced upon**larval feeding of *CYP6B6* dsRNA in *Helicoverpa armigera* (Zhang et al., 2013). In *Spodoptera litura*, the present research shows that silencing *CYP6AB14 *by injecting dsRNA reduced mRNA levels and increased mortality rates (Wang et al., 2015). Silencing of the CYP6 gene affects the growth and development of *Scirpophaga incertulas* (Kola et al., 2016). In *Plutella xylostella*, knockdown of CYP6BG1 by injecting dsRNA dramatically increased the toxicity of chlorantraniliprole by 26.8% 48 h after injection. Notably, when Acc301A1, Acc303A1, or Acc306A1 was silenced, several stress response-related genes were increased (**Figure 6**). These results also show that the three P450 genes play critical roles in the response to pesticide stress in *A. c. cerana*. The results of the study clearly show that injection with dsRNA targeting *Acc301A1*, *Acc303A1*,**and *Acc306A1 *has detrimental effects on workers treated with pesticides.

The supergene family of P450s plays important roles in detoxification, including endogenous and exogenous agents. Combining the results of the enzyme assays, gene expression quantification, and RNAi, we provide evidence to suggest that the four genes were involved in honeybee resistance to the four insecticides, which are usually used in agriculture to defend against ruderal or injurious insects. The different expression profiles of the four genes suggested that P450s might be involved in the resistance to various insecticides. These data verified that proteins encoded by the four P450 genes might function to contribute to insecticide resistance. In summary, this study provides insights into the potential functions of P450s in resistance to insecticides. However, the resistance mechanisms of the four P450 genes in specific insecticides are still unknown. Thus, further functional research on the four P450 genes is essential for their functions in insecticide resistance.

## Data Availability Statement

The raw data supporting the conclusions of this manuscript will be made available by the authors, without undue reservation, to any qualified researcher.

## Ethics Statement

Animal housing facilities and handling protocols were approved by the Animal Welfare and Health Committee of Shandong Agricultural University.

## Author Contributions

BX: designed the experiments and wrote the paper. WZ: carried out the experimental work and wrote the paper YY: participated in the SDS-PAGE and Western blot work. HW: designed the primers and analyzed the data of Western blot. ZL: analyzed the data of RT-qPCR. LM: RNA extraction and cDNA synthesis. YW: carried out the breeding of *Apis cerana cerana*. All authors read and approved the final manuscript

## Funding

This work was financially supported by the Funds of Shandong Province “Double Tops” Program (2016–2020), the National Natural Science Foundation of China (No. 31572470), the earmarked fund for the China Agriculture Research System (No. CARS-44), and Shandong Province Agricultural Fine Varieties Breeding Projects (2017LZN006).

## Conflict of Interest

The authors declare that the research was conducted in the absence of any commercial or financial relationships that could be construed as a potential conflict of interest.

## Abbreviations

P450s, Cytochrome P450 monooxygenases; RT-qPCR, Real-time quantitative PCR; RNAi, RNA interference; A. c. cerana, Apis cerana cerana Fabricius; GSTs, glutathione S-transferases; dsRNA, double-stranded RNA; L1–L5, from the first to fifth instars; Pp, pre-pupae; Pw, white-eyed pupae; Pb, brown-eyed pupae; Pd, dark-eyed pupae; NE, newly emerged; r.h., relative humidity; PBS, phosphate-buffered saline.
