# Supplementary figures and images for: The Roles of Four Novel P450 Genes in Pesticides Resistance in Apis cerana cerana Fabricius: Expression Levels and Detoxification Efficiency
Source: Front Genet. 2019 Nov 15;10:1000. doi: 10.3389/fgene.2019.01000 (PMC6873825; doi:10.3389/fgene.2019.01000)

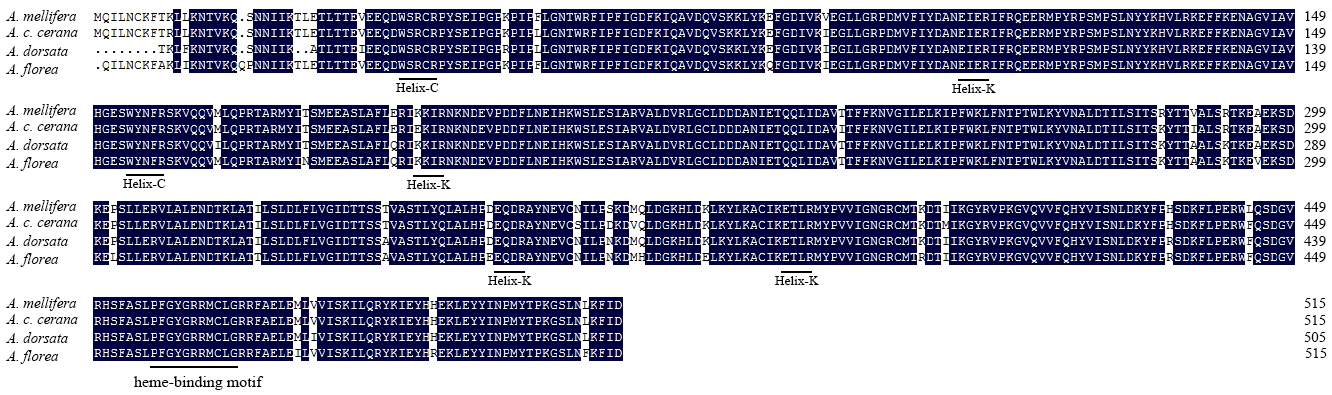

Supplement: Supplementary file 1 [file Image_1.tif]

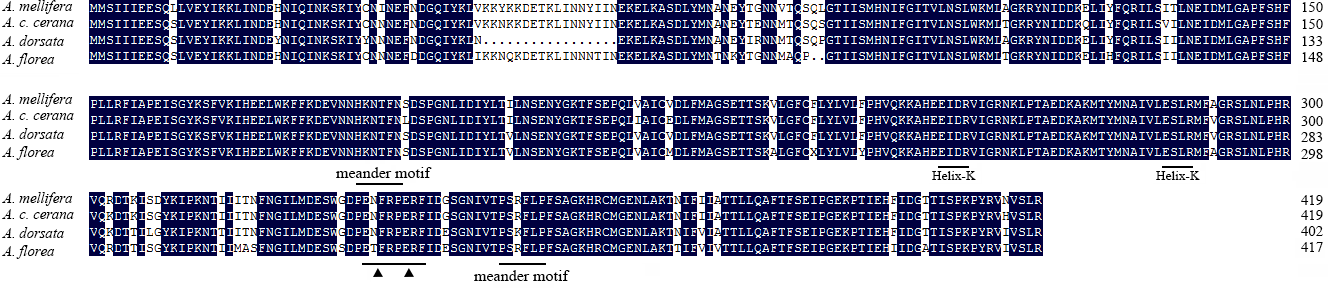

Supplement: Supplementary file 2 [file Image_2.tif]

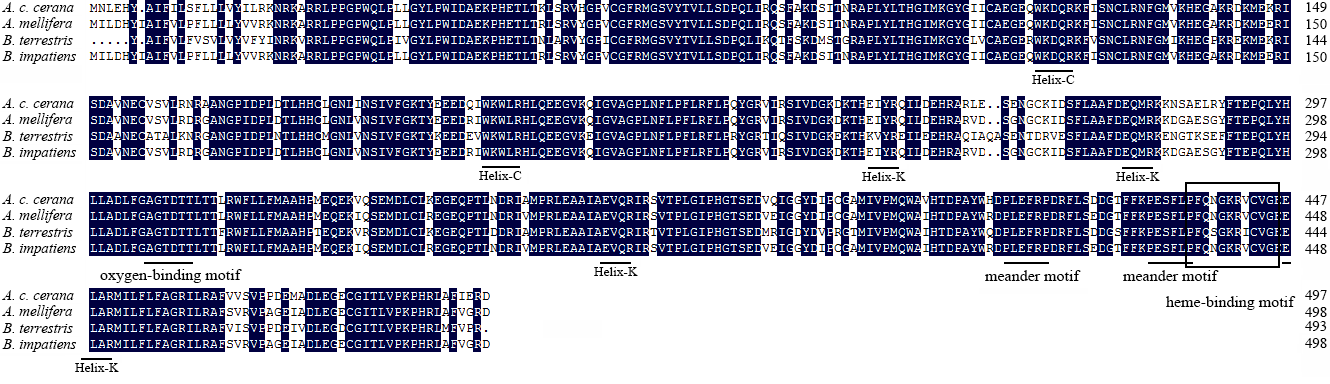

Supplement: Supplementary file 3 [file Image_3.tif]

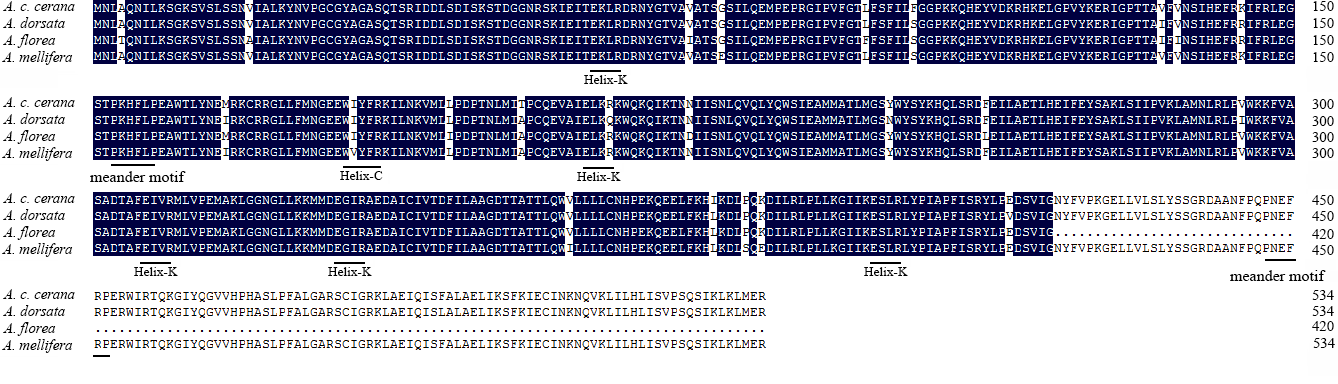

Supplement: Supplementary file 4 [file Image_4.tif]

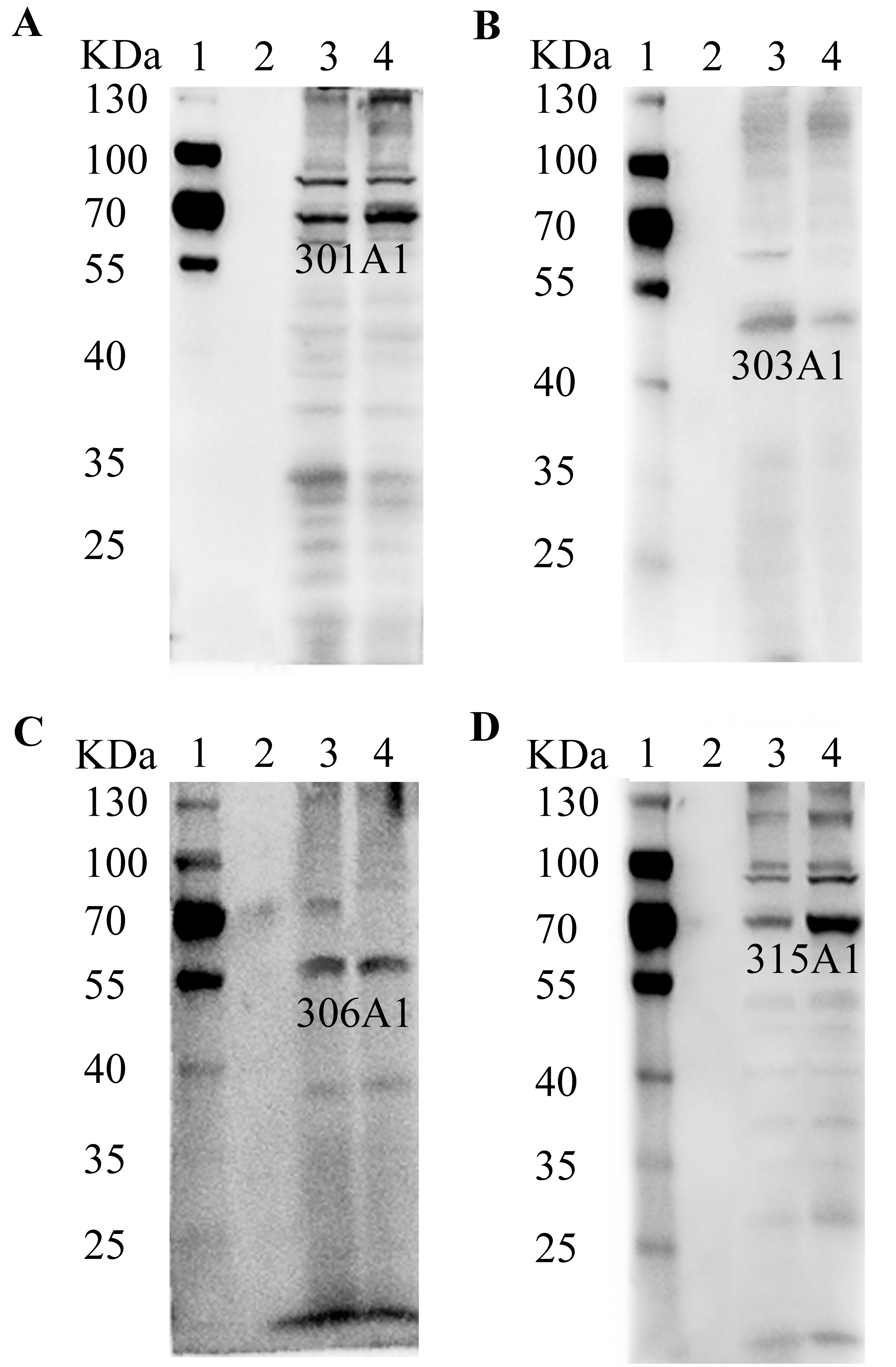

Supplement: Supplementary file 6 [file Image_6.tif]
