# Supplementary material for: The Roles of Four Novel P450 Genes in Pesticides Resistance in Apis cerana cerana Fabricius: Expression Levels and Detoxification Efficiency
Source: Front Genet. 2019 Nov 15;10:1000. doi: 10.3389/fgene.2019.01000 (PMC6873825; doi:10.3389/fgene.2019.01000)
Supplement: Supplementary file 7 [file Table_1.doc]

**Supplementary Table 1.** Abiotic stress conditions for each experimental group.

| Experiment condition | producer and purity | Treatment method | Collection time  after treatment |
| --- | --- | --- | --- |
| Deltamethrin (0.25 mg/L) | Alta Scientific Co., Ltd.; 98%+ | Feed | 0, 3, 6, 12 and 24 h |
| Paraquat (0.25 mg/L) | Alta Scientific Co., Ltd.; 98%+ | Feed | 0, 3, 6, 12 and 24 h |
| DDV (8 mg/L) | Alta Scientific Co., Ltd.; 98%+ | Feed | 0, 3, 6, 12 and 24 h |
| Thiamethoxam (0.1 mg/L) | Aladdin; 98%+ | Feed | 0, 3, 6, 12 and 24 h |
