# Supplementary material for: The Roles of Four Novel P450 Genes in Pesticides Resistance in Apis cerana cerana Fabricius: Expression Levels and Detoxification Efficiency
Source: Front Genet. 2019 Nov 15;10:1000. doi: 10.3389/fgene.2019.01000 (PMC6873825; doi:10.3389/fgene.2019.01000)
Supplement: Supplementary file 8 [file Table_2.doc]

**Supplementary Table 2. Primers for full-length cDNA subcloning, RT-qPCR analysis and dsRNA primers sequence of *Acc301A1*, *Acc303A1*, *Acc306A1* and *Acc315A1* genes.**

| **Gene** | **Applicatiom** | **Primer sequence (5’-3’)** | **Coefficient**  **(R^2^)** | **Product size**  **(bp)** |
| --- | --- | --- | --- | --- |
| *CYP301A1* | cDNA amplication | F: CATTACCTATCAATAAATTTTAAATTTAAGG |  | 1551 |
|  |  | R: ATGCAAATTTTAAATTGTAAATTCACTAG |  |  |
|  | RT-qPCR | F: AAGTGTGCAGCATCCTTCCG | 0.998 | 125 |
|  |  | R: GAACGCCTTTTGGTACGCGA |  |  |
|  | dsRNA^a^ | F: TAATACGACTCACTATAGGGCGAGGTCACGGTGTCG |  | 519 |
|  |  | R: TAATACGACTCACTATAGGGCGACTTATTACGTATTTTCTCG |  |  |
|  | 5’-flanking region | F0: CGCACTTGTCTCGAACGAAAG |  | 1801 |
|  |  | R0: TCGGAATAAGGACGACACCG |  |  |
|  |  | F1: TCAAAAGTGGTTCGTCGTGC |  |  |
|  |  | R1: GACGACACCGGACCAATCT |  |  |
| *CYP303A1* | cDNA amplication | F1: ATGATGTCAATAATAATAGAGGAGAGTC |  | 1263 |
|  |  | R1: TTATTCTATTTCTGGAGATAATTATATTCTTA |  |  |
|  | RT-qPCR | F: GCTTTGTCTTCCGCAGTTGG | 0.987 | 123 |
|  |  | R: TGCGAGGACTTATTCATGGCAG |  |  |
|  | dsRNA^a^ | F: TAATACGACTCACTATAGGGCGATTAATCAATGATG |  | 505 |
|  |  | R: TAATACGACTCACTATAGGGCGAGACTTTGAAAAATTTC |  |  |
|  | 5’-flanking region | F0: AAGTACATCAACTGGCATTCAAC |  | 2038 |
|  |  | R0: CAACGAGTGATGACTCTCCTC |  |  |
|  |  | F1: ACAACTTTGGAAAACTTGCTTGAG |  |  |
|  |  | R1: CGAGTGATTGACTCTCCTCTATT |  |  |
| *CYP306A1* | cDNA amplication | F: ATGAATCTCGAACATTACGCG |  | 1497 |
|  |  | R: TCATCGATCCCGCTCGATAAA |  |  |
|  | RT-qPCR | F: TATCGATACGACGTCCAGCACA | 0.998 | 127 |
|  |  | R: CATCGAGTTGCACATCTTTGTCC |  |  |
|  | dsRNA^a^ | F: TAATACGACTCACTATAGGGCGACAACTTCCGCTCC |  | 484 |
|  |  | R: TAATACGACTCACTATAGGGCGAGCCCAAGCAGTGG |  |  |
| *CYP315A1* | cDNA amplication | F: ATGAATCTTGCGCAAAATATTTTG |  | 1608 |
|  |  | R: TAAATGATATTTCACTTTGTTATATTAGCTA |  |  |
|  | RT-qPCR | F: GTACCTGGTTGCGGTTACGC |  |  |
|  |  | R: CGATCGATTCCCGCCATCTG |  |  |
|  | dsRNAa | F:TAATACGACTCACTATAGGGCGACCGTAAGTCTTTCC |  | 467 |
|  |  | R:TAATACGACTCACTATAGGGCGAGGTGTTGATCCTTC |  |  |
| GFP | dsGFP^a^ (U87974) | F:TAATACGACTCACTATAGGGCGAAGTGGAGAGGGTGAAGGTGA |  |  |
|  |  | R:TAATACGACTCACTATAGGGCGAGGTAAAAGGACAGGGCCATC |  |  |
| *AccTrx1* | RT-qPCR (JX844652) | F:GGTGGTCTTGGTGAAATGAAC  R:CTAAACGCAAAGTCTCATCAACAG | 0.989 |  |
| AccsHSP22.6 | RT-qPCR (KF150016) | F:CGATGAGCACGGTTGGATTTCAC  R:GGTTCTGCTGCTGTTTGGGTG | 0.999 |  |
| *AccGSTO1* | RT-qPCR ( KF496073) | F:CATTCTTTCATGGTAATTCTCCTGGC  R:TTAATCAGTAATCAAATCATATTGTGG | 0.999 |  |
| *β-acting* | RT-qPCR ( XM_017065464) | F:TTATATGCCAACACTGTCCTTT  R:AGAATTGATCCACCAATCCA | 0.998 |  |

^a^ Small letter sequences in primers for dsRNA production are added adaptors containing T7 polymerase promoters.
