# Supplementary material for: The Roles of Four Novel P450 Genes in Pesticides Resistance in Apis cerana cerana Fabricius: Expression Levels and Detoxification Efficiency
Source: Front Genet. 2019 Nov 15;10:1000. doi: 10.3389/fgene.2019.01000 (PMC6873825; doi:10.3389/fgene.2019.01000)
Supplement: Supplementary file 9 [file Table_3.docx]

**Supplementary Table 3.** PCR amplification conditions.

| Primers pair | Amplification conditions |
| --- | --- |
| 301A1-up/dw | 10min at 94℃，40s at 94℃, 40s at 49℃, 60s at 72℃ for 35 cycles, 10 min at 72℃ |
| 303A1-up /dw | 10min at 94℃，40s at 94℃, 40s at 55℃, 70s at 72℃ for 35 cycles, 10 min at 72℃ |
| 306A1-up /dw | 10min at 94℃，40s at 94℃, 40s at 54℃, 70s at 72℃ for 35 cycles, 10 min at 72℃ |
| 315A1-up/dw | 10min at 94℃，40s at 94℃, 40s at 53℃, 70s at 72℃ for 35 cycles, 10 min at 72℃ |
| 301A1-F0/R0 | 10min at 94℃，40s at 94℃, 40s at 55℃, 70s at 72℃ for 35 cycles, 10 min at 72℃ |
| 301A1-F1/R1 | 10min at 94℃，40s at 94℃, 40s at 52℃, 70s at 72℃ for 35 cycles, 10 min at 72℃ |
| 303A1-F0/R0 | 10min at 94℃，40s at 94℃, 40s at 51℃, 70s at 72℃ for 35 cycles, 10 min at 72℃ |
| 303A1-F1/R1 | 10min at 94℃，40s at 94℃, 40s at 53℃, 70s at 72℃ for 35 cycles, 10 min at 72℃ |
| dsRNA-301A1-up/dw | 10min at 94℃，40s at 94℃, 40s at 55℃, 70s at 72℃ for 35 cycles, 10 min at 72℃ |
| dsRNA-303A1-up/dw | 10min at 94℃，40s at 94℃, 40s at 56℃, 40s at 72℃ for 35 cycles, 10 min at 72℃ |
| dsRNA-306A1-up/dw | 10min at 94℃，40s at 94℃, 40s at 57℃, 40s at 72℃ for 35 cycles, 10 min at 72℃ |
| GFP-up/dw | 10min at 94℃，40s at 94℃, 40s at 58℃, 40s at 72℃ for 35 cycles, 10 min at 72℃ |

F0/R0 and F1/R1: primers were used for cloning the 5’-flanking region.
