# Supplementary material for: The Roles of Four Novel P450 Genes in Pesticides Resistance in Apis cerana cerana Fabricius: Expression Levels and Detoxification Efficiency
Source: Front Genet. 2019 Nov 15;10:1000. doi: 10.3389/fgene.2019.01000 (PMC6873825; doi:10.3389/fgene.2019.01000)
Supplement: Supplementary file 10 [file Table_4.docx]

**Supplementary Table 4.** The analysis of the three tested reference genes.

| Gene | GenBank no. | Primer sequence (5’-3’) | R^2^ | E (%) | C.V. (%Ct) |
| --- | --- | --- | --- | --- | --- |
| *β*-actin | XM_017065464 | TTATATGCCAACACTGTCCTTT | 0.998 | 103.5 | 5.01 |
|  |  | AGAATTGATCCACCAATCCA |  |  |  |
| GAPDH | XM_393605 | GATGCACCCATGTTTGTTTG | 0.907 | 71 | 8.51 |
|  |  | TTTGCAGAAGGTGCATCAAC |  |  |  |
| RPS18 | XM_625101 | GATTCCCGATTGGTTTTTGA | 0.846 | 95 | 7.62 |
|  |  | CCCAATAATGACGCAAACCT |  |  |  |

R^2^: correlation coefficient;

E (%): calculated by the standard curve method;

C.V. (%Ct): coefficient of variance expressed as a percentage on the Ct level.
