# Supplementary material for: The Roles of Four Novel P450 Genes in Pesticides Resistance in Apis cerana cerana Fabricius: Expression Levels and Detoxification Efficiency
Source: Front Genet. 2019 Nov 15;10:1000. doi: 10.3389/fgene.2019.01000 (PMC6873825; doi:10.3389/fgene.2019.01000)
Supplement: Supplementary file 11 [file Table_5.docx]

| Gene | cDNA length  (bp) | Amino acids  (AA) | Theoretical  PI | Moleclar weight  (MW, kDa) | predicted peptide antigen |
| --- | --- | --- | --- | --- | --- |
| Acc301A1 | 1551 | 516 | 8.66 | 60.20 | ALSRTKEAEKSD |
| Acc303A1 | 1263 | 420 | 7.67 | 48.38 | RMFVGRSLNLPHRVQKDTK |
| Acc306A1 | 1497 | 498 | 6.77 | 57.08 | GWVKHEGAKRDKMEKRISD |
| Acc315A1 | 1608 | 535 | 9.40 | 60.87 | GASQTSRIDDLSDISKSTDGGN |

**Supplementary Table 5.** The characteristic of *Acc301A1*, *Acc303A1*, *Acc306A1* and *Acc315A1* genes
